# Clinical and Prognostic Implications of *P21* (WAF1/CIP1) Expression in Patients with Esophageal Cancer: A Systematic Review and Meta-Analysis

**DOI:** 10.1155/2020/6520259

**Published:** 2020-01-07

**Authors:** Junbo Wu, Liang Liu, Feng Wu, Li Qiu, Ming Luo, Qing Ke, Xinzhou Deng, Zhiguo Luo

**Affiliations:** ^1^Department of Clinical Oncology, Taihe Hospital, Hubei University of Medicine, Shiyan 442000, China; ^2^Department of Radiation Oncology, Fudan University Shanghai Cancer Center, Shanghai 200032, China; ^3^Department of Oncology, Shanghai Medical College, Fudan University, Shanghai 200032, China

## Abstract

**Background:**

Previous studies have demonstrated that *P21* (WAF1/CIP1) is a valuable prognostic factor in several malignant tumors. However, it is not known whether *P21* can predict the prognosis in patients with esophageal cancer (EC). The aim of this research was to investigate the contribution of *P21* expression to the clinicopathological characteristics and of EC.

**Methods:**

A systematic review and meta-analysis of study focusing on *P21* expression, clinicopathological characteristics, and clinical outcomes in patients with EC was performed using seven databases (PubMed, Embase, Web of Science, and four Chinese databases). Pooled hazard ratios and odds ratios were used to explore the association between *P21* expression, clinicopathological characteristics, and outcomes in patients with EC. The heterogeneity of the studies was classified by the *I*^2^ statistic. The sensitivity analysis was then utilized to assess the robustness of the results. Finally, the funnel plot and Begg's test were used to evaluate the publication bias.

**Results:**

Forty-five studies with 3098 patients were eligible for inclusion in the meta-analysis. Thirty of these studies reported on clinicopathological characteristics and 15 on clinical outcomes. The pooled hazard ratio of 1.456 (95% confidence intervals 1.033–2.053, *P* = 0.032) for overall survival indicated that a low *P21* expression level was an unfavorable prognostic factor for a clinical outcome in patients with EC. Furthermore, the pooled odds ratio confirmed an association between decreased *P21* expression and poor clinicopathological characteristics, including differentiation, lymph node metastasis, invasion, and higher grade and clinical stage. Notably, high *P21* expression was a significant predictor of a favorable response to chemotherapy. There was no evidence of publication bias.

**Conclusion:**

Reduced *P21* expression is associated with a poor outcome in patients with EC.

## 1. Introduction

Esophageal cancer (EC) is the seventh leading cause of cancer mortality worldwide and in 2016 accounted for 15,690 deaths in the United States alone [1]. EC is a complex disease that includes squamous cell carcinoma, adenocarcinoma, and other rarer histologic types. Risk factors are slightly different between the two major types but include sex, race, alcohol consumption, diet, and genetics [2–4]. Several genetic biomarkers are effective in predicting the prognosis of patients with EC, including *TP53*, *CYCLIN D1*, *VEGF*, *COX-2*, and *HER-2* [5]. Moreover, treatment based on these molecular targets has improved survival outcomes in patients with this disease. For example, inhibitors of *c-MET* [6], *EGFR* [7], *HER2* [8], and *VEGR* [9] have been demonstrated to extend survival in these patients. However, drug resistance remains a major concern, and not all patients benefit from targeted therapy. Therefore, novel biomarkers are required to provide insight into the molecular mechanism of EC, identify novel diagnostic methods, and increase the number of treatment options available.

*P21* (WAF1/CIP1), a member of the *P21/P27/P57* family, is a universal cell cycle inhibitor regulated by *P53*. *P21* plays an essential role in the control of cell growth, terminal differentiation, stem cell phenotypes, apoptosis, and cellular stress response. *P21* has also been reported to participate in the proliferation of all types of cells. The expression of *P21* is altered by wild-type *P53* when DNA is damaged, resulting in cell cycle arrest or apoptosis at the G1 checkpoint. *P21* plays a vital role in limiting proliferation and tumor growth, and abnormal expression of this gene has been observed in various types of malignancy. Recent research by Xie and colleagues [10] suggests that overexpression of *P21* is associated with a poor prognosis in patients with non-small-cell lung cancer, while the loss of *P21* protein expression could be a significant predictor of disease progression in patients with pancreatic cancer [11]. A further study demonstrated that aberrant expression of the P21 protein is associated with vascular invasion, pathological disease stage, and overall survival in patients with gastric cancer [12]. Interestingly, Goan et al. reported that overexpression of *P21* predicted an unfavorable survival outcome in patients with esophageal squamous cell carcinoma [13] while other researchers found a significant association of low *P21* expression with shorter survival in patients with the disease [14, 15]. Furthermore, *P21* was found to regulate apoptosis in acute myeloid leukemia cells and malignant glioma cells [16, 17]. Thus, although there is an association of *P21* expression with various types of cancer, the impact of the *P21* level on the disease progression and prognosis of EC remains controversial. Therefore, we performed a systematic review and meta-analysis to assess the potential contribution of *P21* expression to the clinicopathological characteristics and prognosis of EC.

## 2. Method and Materials

### 2.1. Search Strategy

The PubMed, Embase, Web of Science, China National Knowledge Infrastructure, Chongqing VIP, SinoMed, and Wanfang databases were electronically searched up to 30 September 2019. The following search terms were used: (((((((((((((*P21*) OR CIP1) OR SDI1) OR WAF1) OR CAP20) OR CDKN1) OR CDKN1A) OR *P21*CIP1) OR MDA-6)) OR *P21*WAF1)) OR “cyclin-dependent kinase inhibitor *P21*”[Mesh])) AND ((“esophageal neoplasms”[MESH]) OR (((((esophageal cancer) OR esophageal carcinoma) OR esophageal tumor) OR esophageal malignan^∗^) OR esophageal neoplas^∗^).

### 2.2. Inclusion and Exclusion Criteria

Studies were eligible for inclusion in the meta-analysis if they met the following criteria: (1) the subjects were patients diagnosed with any type of EC; (2) *P21* expression in tissue or serum was detected by Western blot, quantitative real-time polymerase chain reaction (PCR), immunohistochemistry, or RNA sequencing; (3) the association of the *P21* expression level with clinicopathological characteristics or the prognosis of EC was investigated; (4) the study population included more than 20 patients with EC; and (5) publication was written in the Chinese or English language. The following exclusion criteria were applied: publication as a review, abstract, experimental study, or letter and no key data provided for the evaluation of the relationship between differential expression of *P21* and the clinicopathological characteristics and survival outcomes in patients with EC.

### 2.3. Data Extraction and Quality Assessment

The following data were collected and tabulated: the surname of the first author, year of publication, histologic type, sample size, country, specimen type, *P21* detection assay used, and the Newcastle-Ottawa Scale (NOS) score. The NOS score was used to assess the quality of the included studies as follows: >6, high quality; 5–6, medium quality; and <5, low quality.

### 2.4. Statistical Analysis

The pooled hazard ratio (HR) and 95% confidence intervals (CIs) were used to estimate the impact of *P21* expression level on the survival outcome in patients with EC. The individual HRs and 95% CIs were extracted directly from the text by two investigators (JW and LL). A pooled HR > 1 and 95% CIs that did not overlap indicated a positive association between a lower *P21* expression level and a poorer survival outcome. When the HR and 95% CIs for survival were not provided, estimates were calculated from the Kaplan-Meier curves according to the method described by Tierney et al. [18]. All data were extracted by two of the authors working independently (FW and LQ). The pooled ORs and associated 95% CIs were used to determine the association between the *P21* expression level and the clinicopathological characteristics of patients with EC according to specimen type (tumor sample vs. normal control), age (younger vs. older), sex (male vs. female), differentiation (poor vs. well or moderate), tumor stage (III–IV vs. I–II), distant metastasis (yes vs. no), lymph node metastasis (yes vs. no), grade (G3–4 vs. G1–2), depth of invasion (III–IV vs. I–II), tumor size (large vs. small), tumor location (upper-middle vs. low), and clinical stage (III/IV vs. I/II). We also explored the relationship between *P21* expression and other better-studied biomarkers of EC, including *P53* and the apoptosis index. The ability of the *P21* level to predict the efficacy of chemotherapy was analyzed by combining the ORs. As with the HRs, an OR > 1 indicated a positive correlation between decreased *P21* expression and poor clinicopathological characteristics.

The *I*^2^ statistic was used to classify the heterogeneity of the studies as low (*I*^2^ < 30%), moderate (30% ≤ *I*^2^ < 60%), substantial (61% ≤ *I*^2^ < 75%), or high (*I*^2^ ≥ 75%) [19]. A *P* value for the *I*^2^ statistic less than 0.10 or *I*^2^ larger than 50% was defined as having statistically significant heterogeneity, and thus, a random-effect model was used. In contrast, a fixed-effect model was used when heterogeneity was not significant. Publication bias was quantified by Begg's test and funnel plot analyses [20]. All statistical analyses were performed using Stata (version 12, StataCorp, College Station, TX, USA).

## 3. Results

### 3.1. Eligible Studies

**Figure 1:**
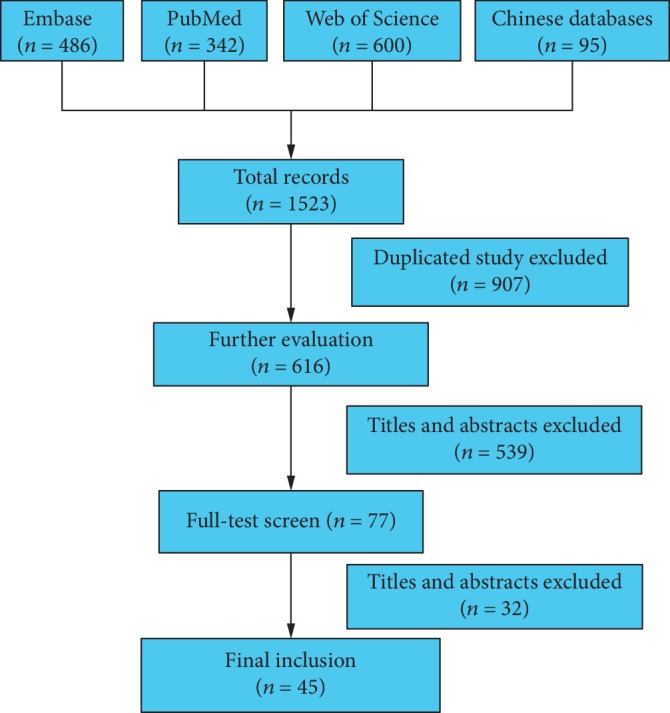
Flowchart of the study selection.

The literature search yielded 1523 citations in total. After removing 907 duplicates, 606 articles were deemed eligible for further evaluation. After screening the titles and abstracts, a further 539 studies were excluded, leaving 74 articles for full-text review. Finally, 45 studies involving 3098 patients with EC were included in the meta-analysis (Figure 1) [13–15, 21–62]. All studies were published between 1997 and 2016 and assessed the correlation between abnormal *P21* expression and outcomes in patients with EC (Table 1). Thirty studies focused on the association of the *P21* expression level with clinicopathological characteristics, and 15 assessed the ability of the *P21* expression level to predict overall survival (Table 2). Twenty-seven studies were performed in China and 11 in Japan. Most of the included studies detected the *P21* level by immunohistochemistry with cutoff values ranging from 1% to 50%, while the remaining studies used real-time PCR or Western blotting. Six studies with a score of 9 and 13 studies with a score of 8 were considered high quality, and 7 studies with NOS scores < 7 were considered low quality.

### 3.2. Prognostic Value of *P21* in Patients with EC

**Figure 2:**
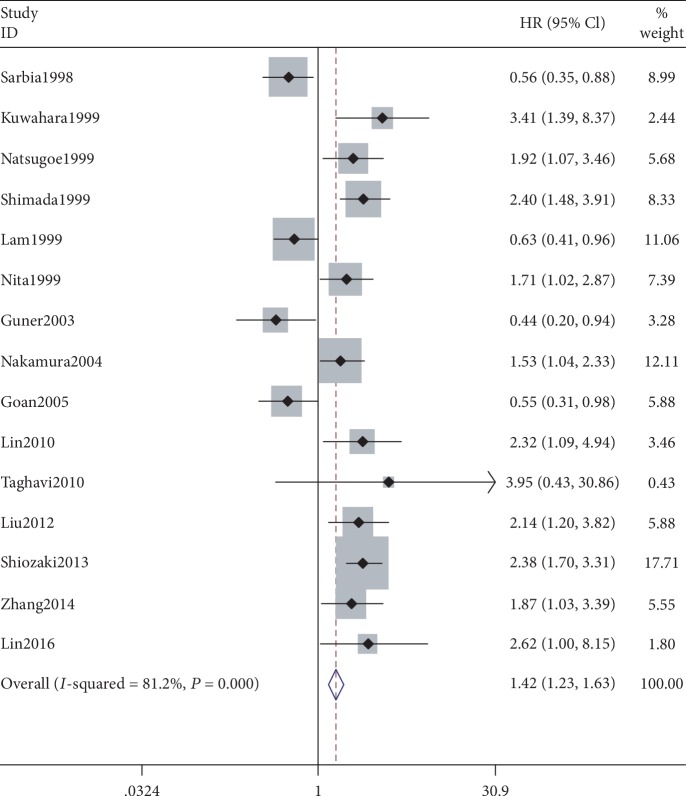
Meta-analysis comparing *P21* expression and overall survival in esophageal cancer patients in 15 studies reporting prognosis of esophageal cancers.

A meta-analysis of the 15 studies that reported overall survival yielded a pooled HR of 1.456 (95% CI: 1.033–2.053, *P* = 0.032, *z* = 2.14), indicating a significant association between the *P21*-negative group and decreased survival time when compared with the *P21*-positive group. Significant heterogeneity was noted across the studies (*I*^2^ = 81.2%, *P* < 0.05, Figure 2). Therefore, a random-effects model was used. Next, subgroup analyses of publication country, continent, sample size, cutoff value, and the statistical methods used to calculate the HRs were performed to explore the origin of the heterogeneity Ultimately, the country of publication might be a source of heterogeneity. The pooled HR of 2.05 indicated that a low *P21* expression level was correlated with shorter survival time in the Japanese studies. Notably, the degree of heterogeneity in this group was reduced in the fixed-effects model (*I*^2^ = 0.0%, *P* = 0.425). However, obvious heterogeneity was also detected in the other subgroup analyses.

### 3.3. Correlation between *P21* and Clinicopathological Characteristics of Patients with EC

**Figure 3:**
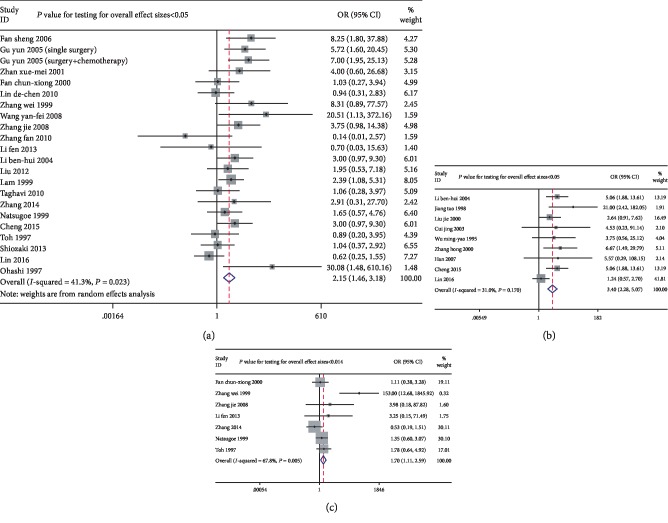
Forest plots of odds ratios for *P21* expression and clinicopathological parameters including differentiation, grading, and clinical stage in esophageal cancer patients: (a) differentiation (OR = 2.153, 95% CIs: 1.455-3.184, *P* < 0.05); (b) grading (OR = 3.399, 95% CIs: 2.278-5.071, *P* < 0.05); (c) clinical stage (OR = 1.697, 95% CIs: 1.111-2.594, *P* = 0.014).

**Figure 4:**
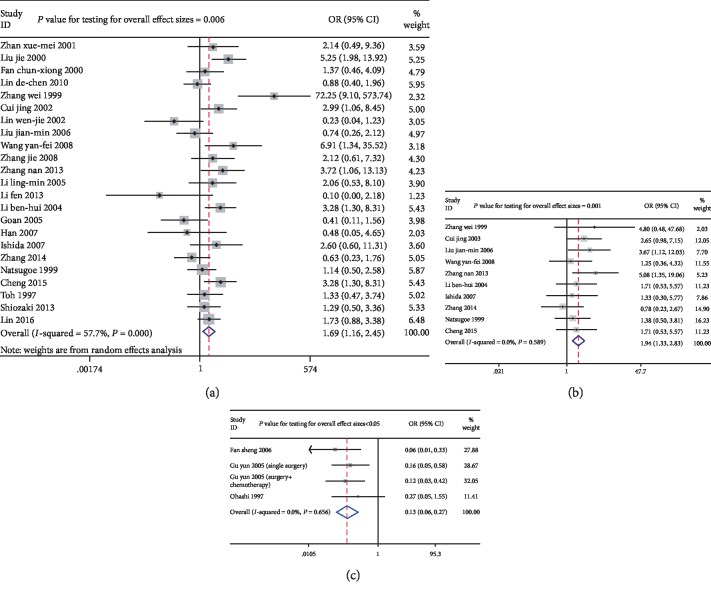
Forest plots of odds ratios for *P21* expression and clinicopathological parameters including lymph node metastasis, invasion, and apoptosis index in esophageal cancer patients: (a) lymph node metastasis (OR = 1.691, 95% CIs: 1.165~2.455, *P* = 0.006); (b) invasion (OR = 1.939, 95% CIs: 1.328-2.830, *P* = 0.001); (c) apoptosis index (OR = 0.131, 95% CIs: 0.064-0.269, *P* < 0.05).

**Figure 5:**
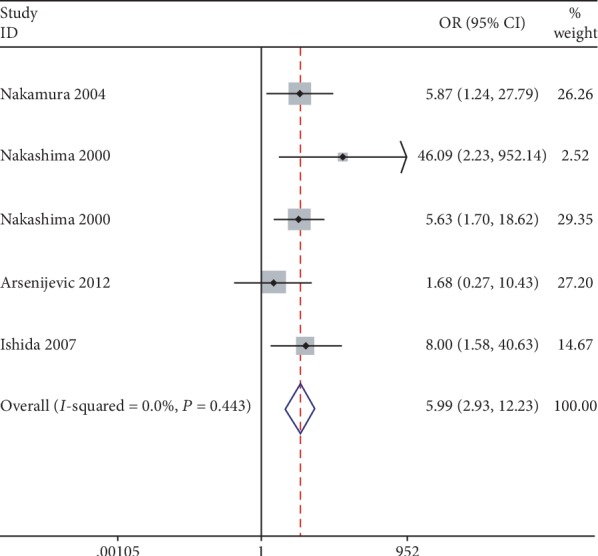
Forest plots of odds ratios for *P21* expression and clinicopathological parameters including chemotherapy effectivity in esophageal cancer patients.

Decreased *P21* expression was observed in tumors with poorer differentiation (pooled OR = 2.153, 95% CIs 1.455–3.184). Significant heterogeneity was found between the studies (*I*^2^ = 41.30%, *P* = 0.023, Figure 3(a)), so a random-effect model was used. There was a significant association of lower *P21* expression with a higher tumor grade (pooled OR = 3.399, 95% CI 2.278–5.071, *P* < 0.05, *z* = 5.99). No significant heterogeneity was found between the studies (*I*^2^ = 31.00%, *P* = 0.17, Figure 3(b)). Significant heterogeneity was found between the studies reporting on the clinical stage (*I*^2^ = 53.4% and *P* = 0.002), so a random-effect model was used. There was a significant correlation between decreased *P21* expression and an advanced clinical stage (pooled OR = 1.697, 95% CI 1.111–2.594, *P* = 0.014, Figure 3(c)). There was also a significant correlation between *P21* expression and lymph node metastasis (pooled OR = 1.691, 95% CI 1.165–2.455, *P* = 0.006, *z* = 2.76) in 23 studies, in which there was slight heterogeneity (*I*^2^ = 57.70%, *P* < 0.05, Figure 4(a)). Lower *P21* expression was significantly associated with a higher risk of invasion (pooled OR = 1.939, 95% CI 1.328–2.83, *P* = 0.001; *I*^2^ = 0.00%, *P* = 0.589, Figure 4(b)). Moreover, a significant correlation was found between low *P21* expression and a low apoptosis index (pooled OR = 0.131, 95% CI 0.064–0.269, *P* < 0.05, *z* = 5.55; *I*^2^ = 0.00%, *P* = 0.656, Figure 4(c)). Importantly, there was a significant association between a high *P21* expression level and a favorable response to chemotherapy (pooled OR = 5.987, 95% CI 2.930–12.234, *P* < 0.05, *z* = 4.91; *I*^2^ = 0.00%, *P* = 0.443, Figure 5). However, there was no significant association between *P21* expression and any other clinical parameters (Table 3).

### 3.4. Sensitivity Analysis and Publication Bias

**Figure 6:**
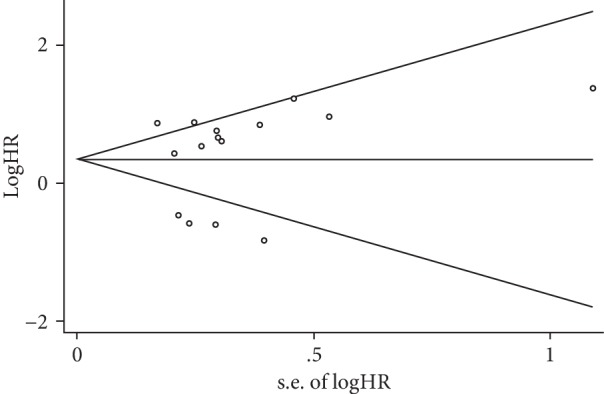
Funnel plot for the publication bias test of the analysis between *P21* expression and overall survival in esophageal cancer patients. The horizontal line means the pooled effect estimate.

A sensitivity analysis confirmed that the results were not obviously impacted by any individual study, suggesting that the meta-analysis had good stability. The results of Begg's test and the funnel plot did not indicate any publication bias (Figure 6, Table 3).

## 4. Discussion

There is accumulating evidence to suggest that abnormal expression of *P21* is present in various types of malignancy, including gastric [63], lung [64], and tonsillar [65] cancers. However, the results of studies that have investigated the potential role of *P21* expression are not consistent. To our knowledge, this meta-analysis contains the largest number of studies that have evaluated the association between *P21* expression and the clinicopathological characteristics and outcomes in patients with EC. We found that overall survival in patients with EC was likely to be longer in those with higher *P21* expression than in those with lower *P21* expression. Our finding that decreased *P21* expression was correlated with disease progression, that is, differentiation, lymph node metastasis, and invasion, as well as an advanced disease grade and clinical stage, indicates that *P21* has a suppressor role in EC. A particularly important finding in this study was that *P21* might be a valuable predictor of the effectiveness of chemotherapy.

In this study, there was a significant association between low *P21* expression and a poorer outcome of EC. In contrast, high *P21* expression has been reported to be an unfavorable prognostic factor in patients with prostate cancer [66] and breast cancer [67]. However, the results of yet other studies in patients with cervical adenocarcinoma [68] and bladder cancer [69] are consistent with our finding that *P21* might act as a tumor suppressor. Like in our study, previous research showed significant associations between a low *P21* level and advanced clinical stage and grade of bladder cancer, indicating that *P21* has an important role in tumor progression [70]. A previous study in prostate cancer showed that P21 inhibits cell growth by targeting *E2F1* [71]. It has been confirmed that *P21* expression could be reduced by *DDX3* in lung cancer, leading to inhibition of the growth of cancer cells. Wu and colleagues demonstrated that inhibition of *P21* via the *P53*-*DDX3* pathway may promote the proliferation of cancer cells and tumor growth in vitro and in vivo [69]. Moreover, it has been shown that *P21* interacts with subunits of cyclin-dependent kinases [72], resulting in inhibition of tumor growth and progression. Finally, the tumor suppressor activity of *P21* can be promoted by interaction with tumor-related factors like *MYC*, proliferating cell nuclear antigen (*PCNA*), and signal transducer and activator of transcription (STAT3) [73–76].

This study has several limitations that should be taken into account when interpreting its results. First, according to the NOS criteria, the quality of the included studies was variable (ranging from a score of 6 to a score of 9). Second, several HR values and their respective 95% CIs were obtained from Kaplan-Meier curves, potentially leading to inaccurate results. The inclusion of univariate HRs without adjustment could also have contributed to heterogeneity. Third, the methodological differences between the studies may have resulted in the underestimation of the effect size. For example, most of the studies detected *P21* expression by immunohistochemistry, but some used diverse methods, including Western blot and real-time quantitative PCR. The use of the streptavidin-peroxidase conjugate in some studies and the streptavidin-biotin complex method in others was also a potential source of heterogeneity. Another methodological difference was that the most common cutoff values for the detection of *P21* were 10% and 50%, but this was not completely consistent across the studies. Inclusion of research published only in Chinese or English may have been another source of bias, given that negative results tend to be published in local journals. Furthermore, the number of studies included in this analysis was limited and we only restrict the patient number for the enrolled studies by a threshold of 20.

## 5. Conclusion

In this study, the results suggest that low *P21* expression has a clinically important negative clinicopathological and prognostic impact in patients with EC. Well-designed studies that include larger patient cohorts are required to identify the mechanisms underlying how *P21* is involved in the tumorigenesis and progression of EC.

## Figures and Tables

**Table 1:** Characteristics of studies included in the meta-analysis.

Study and year	Year	Cancer	Sample size	Country	Specimen type	Cutoff value	Method	NOS quality score
Wu 1995	1995	Esophageal cancer	40	China	Tissue	NA	Immunohistochemistry	7
Ohashi 1997	1997	SCC	25	Japan	Tissue	NA	Immunohistochemistry	6
Toh 1997	1997	SCC	61	Japan	Tissue	10%	Immunohistochemistry	7
Jiang 1998	1998	SCC	46	China	Tissue	10%	Immunohistochemistry	7
Sarbia 1998	1998	SCC (surgical treatment)	149	Germany	Tissue	50%	Immunohistochemistry	9
Kuwahara 1999	1999	SCC	32	Japan	Tissue	10%	Immunohistochemistry	8
Lam 1999	1999	SCC	153	Hong Kong	Tissue	50%	Immunohistochemistry	7
Natsugoe 1999	1999	SCC	111	Japan	Tissue	10%	Immunohistochemistry	8
Nita 1999	1999	SCC	62	Japan	Tissue	14	Immunohistochemistry	9
Shimada 1999	1999	SCC	116	Japan	Tissue	50%	Immunohistochemistry	8
Zhang 1999	1999	Esophageal cancer	38	China	Tissue	5%	Immunohistochemistry	9
Fan 2000	2000	Esophageal cancer	56	China	Tissue	NA	Immunohistochemistry	7
Liu 2000	2000	SCC	80	China	Tissue	1%	Immunohistochemistry	8
Nakashida 2000	2000	SCC	30	Japan	Tissue	10%	Immunohistochemistry	7
Matsumoto 2001	2001	SCC	79	Japan	Tissue	10%	Immunohistochemistry	7
Zhan 2001	2001	Esophageal cancer	30	China	Tissue	10%	Immunohistochemistry	6
Li 2002	2002	AD	35	China	Tissue	NA	Immunohistochemistry	6
Cui 2003	2003	SCC	72	China	Tissue	5%	Immunohistochemistry	8
Guner 2003	2003	SCC	63	Germany	Tissue	10%	Immunohistochemistry	7
Zhang 2003	2003	SCC	43	China	Tissue	0%	Immunohistochemistry	7
Li 2004	2004	SCC	80	China	Tissue	25%	Immunohistochemistry	8
Li Li 2004	2004	SCC	48	China	Tissue	NA	Immunohistochemistry, ISH	6
Nakamura 2004	2004	SCC	76	Japan	Tissue	10%	Immunohistochemistry	9
Chang 2005	2005	Esophageal cancer	118	Korea	Tissue	10%	Immunohistochemistry	7
Goan 2005	2005	SCC	36	China	Tissue	50%	Immunohistochemistry	8
Gu 2005	2005	SCC (single surgery)	50	China	Tissue	10%	Immunohistochemistry	7
Gu 2005	2005	SCC (surgery+chemotherapy)	50	China	Tissue	10%	Immunohistochemistry	7
Li 2005	2005	SCC	43	China	Tissue	10%	Immunohistochemistry	9
Fan 2006	2006	SCC	40	China	Tissue	10%	Immunohistochemistry	7
Liu 2006	2006	SCC	60	China	Tissue	25%	Immunohistochemistry	6
Han 2007	2007	SCC	40	Turkey	Tissue	10%	Immunohistochemistry	7
Ishida 2007	2007	SCC	32	Japan	Tissue	20%	Immunohistochemistry	8
Wang 2008	2008	SCC	48	China	Tissue	25%	Immunohistochemistry	8
Zhang 2008	2008	SCC	45	China	Tissue	NA	RT-PCR	7
Lin 2010	2010	SCC	148	China	Tissue	10%	Immunohistochemistry	8
Taghavi 2010	2010	SCC	80	Iran	Tissue	50%	Immunohistochemistry	8
Zhang 2010	2010	SCC	90	China	Tissue	NA	Immunohistochemistry	7
Arsenijevic 2012	2012	SCC	41	Serbia	Tissue	NA	Immunohistochemistry	6
Liu 2012	2012	SCC	189	China	Tissue	NA	Immunohistochemistry	6
Li 2013	2013	SCC	48	China	Tissue	NA	RT-PCR	7
Shiozaki 2013	2013	SCC	69	Japan	Tissue	30%	Immunohistochemistry	8
Zhang 2013	2013	SCC	51	China	Tissue	NA	Western blot	7
Zhang 2014	2014	SCC	62	China	Tissue	50%	Immunohistochemistry	7
Cheng 2015	2015	SCC	80	China	Tissue	25%	Immunohistochemistry	8
Lin 2016	2016	SCC	153	China	Tissue	10%	Immunohistochemistry	9

**Table 2:** The main characteristics of studies investigating the prognostic value of *P21* and overall survival.

Study	HR	LL	UL	Survival	Statistical method
Sarbia M 1998	0.556	0.347	0.885	OS	Multivariable
Kuwahara M 1999	3.409	1.388	8.373	OS	Multivariable
Natsugoe S 1999	1.920	1.065	3.460	OS	Survival curve
Shimada Y 1999	2.398	1.477	3.906	OS	Multivariable
Lam KY 1999	0.627	0.411	0.956	OS	Survival curve
Nita ME 1999	1.713	1.022	2.871	OS	Multivariable
GUNER D 2003	0.435	0.200	0.943	OS	Multivariable
Nakamura T 2004	1.530	1.040	2.330	OS	Multivariable
Goan YG 2005	0.549	0.308	0.980	OS	Multivariable
Lin CD 2010	2.322	1.091	4.940	OS	Univariate
Taghavi N 2010	3.946	0.430	30.860	OS	Multivariable
Liu J 2012	2.139	1.199	3.816	OS	Survival curve
Shiozaki A 2013	2.379	1.700	3.313	OS	Multivariable
Zhang Y 2014	1.867	1.029	3.387	OS	Multivariable
Lin Y 2016	2.623	1.005	8.147	OS	Multivariable

Note: HR = hazard ratio; LL = lower confidence interval limit; UL = upper confidence interval limit; OS = overall survival.

**Table 3:** Main results for meta-analysis between p21 and clinicopathological features in esophageal cancer.

Clinical parameters	OR [95% CI]	*P*	*z*	*I* ^2^	*P*	Begg's test
Tissue	2.210 [0.811-6.022]	0.121	1.550	90.9%	*P* < 0.05	0.373
Age	0.969 [0.716-1.312]	0.840	0.210	81.8%	*P* < 0.05	0.732
Gender	0.758 [0.566-1.015]	0.063	1.860	85.4%	*P* < 0.05	0.484
Differentiation	2.153 [1.455-3.184]	*P* < 0.05	3.840	41.3%	0.023	0.185
Clinical stage	1.616 [1.068-2.446]	0.023	0.002	53.4%	0.002	0.096
Tumor stage	0.885 [0.541-1.448]	0.627	0.490	93.7%	*P* < 0.05	0.089
Lymph node	1.691 [1.165-2.455]	0.006	2.760	57.7%	*P* < 0.05	1.000
Metastasis	0.637 [0.356-1.140]	0.129	1.520	76.7%	*P* < 0.05	0.296
Grading	3.399 [2.278-5.071]	*P* < 0.05	5.990	31.0%	0.170	0.532
Invasion	1.939 [1.328-2.830]	0.001	3.430	58.9%	*P* < 0.05	0.788
Tumor size	1.005 [0.602-1.675]	0.986	0.020	36.0%	0.181	0.806
Tumor location	0.971 [0.553-1.706]	0.918	0.100	51.8%	0.034	0.917
P53	1.700 [0.918-3.148]	0.092	1.690	63.6%	0.003	0.210
AI (apoptosis index)	0.131 [0.064-0.269]	*P* < 0.05	5.550	65.6%	*P* < 0.05	1.000
Chemotherapy effectivity	5.987 [2.930-12.234]	*P* < 0.05	4.910	44.3%	*P* < 0.05	0.462

OR = odds ratio; CI = confidence interval.
